# Supplementary material for: A New Method to Evaluate Lower Esophageal Distension Capacity in Eosinophilic Esophagitis by Using Functional Lumen Imaging Probe (EndoFLIP™)
Source: Diagnostics (Basel). 2024 Jan 19;14(2):218. doi: 10.3390/diagnostics14020218 (PMC10814586; doi:10.3390/diagnostics14020218)
Supplement: Supplementary file 1 [file diagnostics-14-00218-s001.zip › Supplementary Tables.pdf]

Table S1: Esophageal diameters at maximal volume distension and esophageal dynamics in healthy control subjects with normal esophagus, as determined with Endoflip^{TM}

Subject	Diameter [mm]	Max Pressure [mmHg]	Max Volume [mL]	Contractions	Antegrade
C1	17.396	38.85	60	yes	Yes
C2	17.667	44.72	50	yes	Yes
C3	17.098	42.5	60	yes	Yes
C4	16.767	36.1	60	yes	Yes
C5	18.691	34.49	60	yes	No
C6	16.983	64.66	60	yes	Yes
C7	16.496	59.57	60	yes	Yes
C8	19.909	49.17	50	yes	Yes
C9	16.549	48.83	55	yes	Yes
C10	13.312	51.05	60	yes	Yes

Table S2: Esophageal diameters at maximal volume distension and esophageal dynamics in patients with active EoE before treatment (BT) measured with Endoflip^{TM}

Patient	Diameter [mm]	Max Pressure [mmHg]	Max Volume [mL]	Contractions	Antegrade
P1BT	11.135	60.21	60	no	-
P2BT	10.437	63.17	60	no	-
P3BT	12.819	65.41	35	yes	yes
P4BT	15.394	61.62	45	yes	yes
P5BT	15.211	86.28	60	yes	yes
P6BT	17.51	43.37	60	yes	yes
P7BT	12.499	66.18	60	no	-
P8BT	13.391	85.32	60	no	-
P9BT	14.165	50.32	60	yes	yes
P10BT	12.906	77.32	60	no	-
P11BT	16.429	70.77	45	yes	yes
P12BT	18.121	53.15	60	yes	yes
P13BT	13.539	76.39	55	yes	yes
P14BT	19.693	41.49	60	yes	yes

Table S3: Esophageal diameters at maximal volume distension and esophageal dynamics in patients with EoE in remission after treatment (AT) with budesonide, measured with Endoflip^{TM}

Patient	Diameter [mm]	Max Pressure [mmHg]	Max Volume [mL]	Contractions	antegrade
P1AT	15.473	58.59	60	yes	yes
P2AT	13.398	81.82	60	yes	yes
P3AT	15.093	68.99	45	yes	yes
P4AT	16.69	64.26	60	yes	yes
P5AT	15.416	65.45	60	yes	yes
P6AT	17.353	54.16	60	yes	yes
P7AT	14.868	67.92	55	yes	yes
P8AT	15.225	69.09	60	yes	yes
P9AT	18.855	52.84	60	yes	yes
P10AT	14.68	52.12	60	yes	yes
P11AT	18.128	54.02	60	yes	yes
P12AT	19.833	22.04	60	yes	yes
P13AT	14.546	52.9	60	yes	yes
P14AT	17.58	39.45	60	yes	yes
