# Supplementary material for: A New Method to Evaluate Lower Esophageal Distension Capacity in Eosinophilic Esophagitis by Using Functional Lumen Imaging Probe (EndoFLIP™)
Source: Diagnostics (Basel). 2024 Jan 19;14(2):218. doi: 10.3390/diagnostics14020218 (PMC10814586; doi:10.3390/diagnostics14020218)
Supplement: Supplementary file 1 [file diagnostics-14-00218-s001.zip › Supplementary_text_S1.pdf]

Video description: This video shows the basics steps of the procedure proposed in our work.

Step 1: Once the study file is loaded, the first step consists in selecting the record part corresponding to the lower esophagus as usually the whole study encompass not only this esophagus part but alse the middle an upper esophagus.

Step 2: In this step, one should select the fixed-volume steps in the study staircase. As explained in the work, it is important to select those fixed-volume steps in which a monotonic volume-pressure exists. In the particular case of the video, the selected range encompass the fourth fixed-volume corresponding to 35 mL to the nineth fixed-volume corresponding to 60 mL. In this way the first three volume are discarded.

Step 3: In the selected range, the next step involves the selection of the EGJ, that is the approximate channels which record the diameter at the EGJ; this is done by selecting channel-by-channel as shown in the video. Note that the channel selection is always approximate due the low resolution of the channels. In any case, the method always selects for the analysis two channel above the EGJ.

Step 4: During this step the procedure descripted in the work calculates several things. The first one is the correction of the potential displacement of the balloon sensors with respect to the esophageal wall, as explained in the work, in particular in Figure 2 and in the point 2.5. In this way, the subsequent calculation are performed taking into account the diameters of the same esophagus wall during the balloon inflation. Because in the last fixed-volume (at 60 mL) the EGJ is approximately located in channel #8, and we two channel above the EGJ, we always use channels #1 to #6 for fitting the Michaelis Menten curve, that is, six points for every fixed-volume.

OUTCOME: The final result, that is, the calculated diameter at V=60 mL, so-called D(60 mL) Distension Capacity is calculated as the fitted value at that volume, as shown in the upper left plot.
